# A Pitstop-2 analog impairs viability of aggressive lung cancer cells by disrupting nuclear pore integrity

**DOI:** 10.1039/d5na00410a

**Published:** 2025-09-16

**Authors:** Sílvio Terra Stefanello, Caren Rigon Mizdal, Christian Paul Konken, Günter Haufe, Victor Shahin

**Affiliations:** a Institute of Physiology II, University of Münster Robert-Koch-Str. 27b 48149 Münster Germany terraste@uni-muenster.de shahin@uni-muenster.de; b European Institute for Molecular Imaging (EIMI), University of Münster Münster Germany; c Department of Nuclear Medicine, University Hospital Münster Münster Germany; d Organic Chemistry Institute, University of Münster Münster Germany

## Abstract

We previously demonstrated that Pitstop-2, an inhibitor of clathrin-mediated endocytosis (CME), exhibits CME-independent inhibitory effects on nuclear pore complexes (NPCs). Pitstop-2 interferes with β-propeller folds in both clathrin coats and NPC scaffold proteins. NPCs are not only the mediators of all nucleocytoplasmic transport but are also involved in regulating fundamental cellular physiological processes, including gene expression and proliferation. Their upregulation is strongly associated with malignant transformation, as evidenced in our studies involving non-small cell lung cancer (NSCLC) cells. Therefore, herein, we set out to design and synthesize novel compounds using Pitstop-2 as a lead substance. Since the inhibition of NPC formation was recently shown to cause cancer cell death selectively, our efforts focused on designing compounds with enhanced inhibitory effects on NPCs. Among these, a Pitstop-2 analog, CSV-22, demonstrated the highest pharmacological potency and exhibited NPC-disruptive effects superior to those of Pitstop-2 at lower concentrations. Computational docking analysis revealed that CSV-22 interacts with β-folds in NPC scaffold proteins, which are essential for the structural and functional integrity of NPCs. Functional assays revealed that CSV-22 selectively impairs viability in highly metastatic NSCLC cells, with lower IC_50_ values after 24-hour exposure. Transferrin uptake assays further suggest that CSV-22 does not significantly inhibit CME in NSCLC, distinguishing its mechanism from Pitstop-2. These findings position CSV-22 as a promising candidate for targeted cancer therapy.

## Abstract 2

CSV-22, a novel Pitstop-2 analogue, disrupts nuclear pore complexes, impairing nucleocytoplasmic transport and reducing cell viability, particularly in metastatic lung cancer cells.

## Graphical abstract

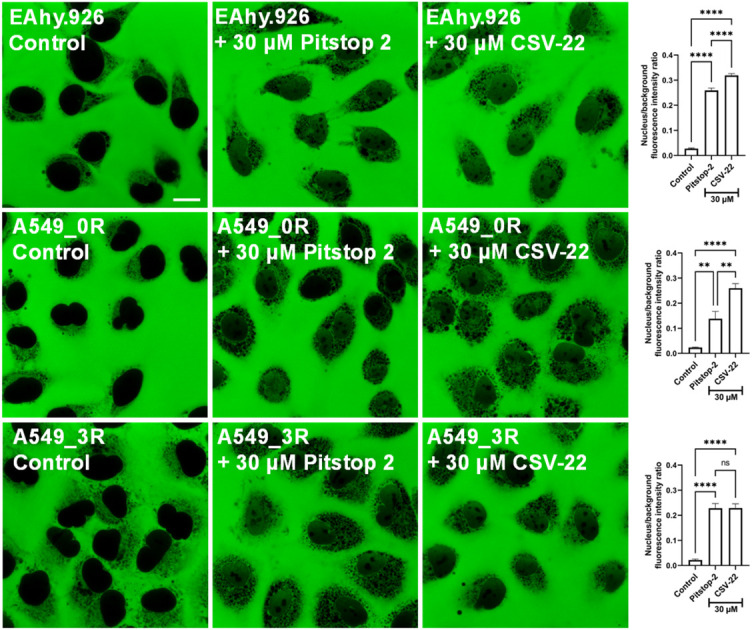

## Introduction

**Fig. 1:**
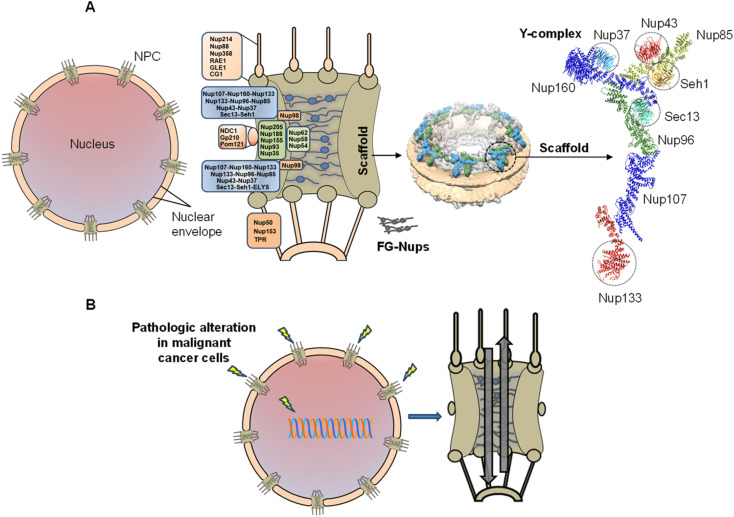
Schematic of the nuclear pore complex (NPC) and pathologic alteration. (A) NPCs span the nuclear envelope. The NPC is composed of different proteins (termed Nups), arranged in sub-complexes making up the NPC scaffold and central channel, which is occupied by FG-Nups. The scaffold is made up of Y-shaped subcomplexes,^9^ termed Y-complexes as shown in cryo-electron tomography analysis.^10^ (B) Alteration of the physiological integrity of NPCs, including substantially increased nucleocytoplasmic transport rate promote malignant transformation and proliferation of cancer cells.

Clathrin-mediated endocytosis (CME) is a fundamental physiological process essential for the exchange of material and communication of a eukaryotic cell with its environment. Significant upregulation of CME is associated with pathophysiologic processes including diverse viral diseases and malignant transformation of cancer cells, a fact that has inspired scientists to develop small molecule compounds capable of inhibiting the clathrin-mediated uptake.^1,2^ Numerous compounds have been presented in recent years,^1,3,4^ suggesting potential therapeutic applications, while still lacking the necessary degree of specificity or a defined mechanism of action. Pitstop-2 is the most recent inhibitor of CME and it has been demonstrated to bind directly to the terminal β-propeller domain of the clathrin heavy chain ultimately disrupting CME by causing an arrest of coated pit dynamics.^2^ Clathrin coats share striking structural similarities with other physiologically crucial cellular components, namely nuclear pore complexes (NPCs), schematically depicted in Fig. 1.^5–8^

NPCs mediate all nucleocytoplasmic exchange of material (Fig. 1). NPCs regulate many physiologically essential processes throughout the lifecycles of cells and tissue, including homeostasis, differentiation, gene expression, and proliferation.^11–16^ Like CME, upregulation of NPC transport is associated with diverse pathologies, including the malignant transformation of cancer cells (Fig. 1), to meet their substantial proliferation rate.^17,18^ Both clathrin coats and NPC scaffold proteins are rich in β-propeller folds.^6,7,19^ Such folds are particularly abundant in NPC scaffold proteins (Fig. 1). The NPC scaffold comprises the Y-shaped Nup107/160 subcomplex with nine members (Fig. 1), six of which have β-propeller domains (Nup37, Nup43, Nup133, Nup160, Seh1, Sec13) crucial for the structural and functional integrity of NPCs.^8,10,20,21^ We have recently shown with computational docking analysis that Pitstop-2 can directly interfere with most of them.^8^ Our theoretical analysis may provide a mechanistic explanation to our previous direct experimental findings regarding Pitstop-2-induced disruption of NPC structure and permeability barrier, based on ultrastructural and permeability investigations with atomic force microscopy and confocal fluorescence microscopy, respectively.^22^

Using Pitstop-2 as a lead substance, we set out to design and synthesize more potent analogous compounds, focusing on NPCs as the target. We aimed to exploit the disruptive effects on NPCs to disrupt highly aggressive NSCLC cells, wherein we recently demonstrated the crucial roles NPCs and their upregulation play in the malignant transformation of cells.^17^ Indeed, aggressive cancer cells are substantially more sensitive to NPC inhibition, as recently demonstrated by Sakuma *et al.*,^18^ who showed that inhibition of NPC formation selectively induces cancer cell death. This sensitivity is largely attributed to the fact that, in malignant cells, nucleocytoplasmic transport *via* NPCs is often hijacked to promote tumor growth and evade apoptosis, further underscoring the central role of NPCs in cancer progression.^23^ From a series of compounds we designed, synthesized, and tested, one stood out, termed CSV-22.

In the present study, we introduce CSV-22 as a novel Pitstop-2 analog, which strongly interacts with the terminal β-propeller domains of NPC scaffold proteins with higher efficacy and binding affinity than Pitstop-2. We demonstrated the superiority of CSV-22 compared to Pitstop-2 by utilizing experimental permeability assays and molecular docking analyses. Additionally, when we exposed highly metastatic NSCLC cells to CSV-22, we found that it reproduced the Pitstop-2 effects at significantly lower concentrations.

## Experimental section

### Cell culture

A549_3R were cultured at 37 °C, 5% CO_2_ in Dulbecco's Modified Eagle's Medium (Invitrogen Corp., Karlsruhe, Germany) supplemented with 10% fetal calf serum (FCS, PAA Clone, Coelbe, Germany). The generation of the highly aggressive A549 cell line (A549_3R) available in our laboratories for years,^17,24^ was described previously.^25^

The lowly metastatic A549 cell line (A549_0R) and EA.hy926 were commercially purchased from ATCC (A549 (ATCC® CCL-185); EA.hy926 (ATCC® CRL-2922)).

### Chemicals and CVS-22 synthesis

Pitstop-2 were purchased from Sigma-Aldrich (St. Louis, MO, USA). Chemical yields were calculated relative to the minor reactant.

For thin-layer chromatography (TLC) readily coated silica (standard or C-18) or neutral alumina PE-plates (POLYGRAM® SIL G/UV254) from MACHEREY-NAGEL with 0.2 mm layer thickness were used. Detection was accomplished using UV-light (wavelength = 254 nm or 366 nm) or using the following reagent solution followed by heating to 250 °C:

- Permanganate-solution (3 g potassium permanganate, 0.25 g sodium hydroxide, 20 g sodium carbonate solved in 300 mL distilled water).

Automated column chromatography utilizing a Reveleris X2 from GRACE (now BÜCHI) was performed. The relevant parameters can be found in form of a table at the experiment.

The NMR spectra were recorded on an Agilent DD2 600 spectrometer. Chemical Shifts are reported on the δ scale relative to tetramethylsilane (0 ppm) or using solvent signals for calibration. ^1^H NMR spectra were referenced relative to the used deuterated solvent, ^13^C NMR spectra were referenced to the used deuterated solvents and are decoupled. Analysis and processing of NMR-spectra was performed using MestReNova (version 14.2.0) from Mestrelab Research S. L.

Mass spectra were recorded on the following device using electrospray ionization (ESI):

- Orbitrap LTQ XL (Thermo Scientific).

**Fig. 2:**
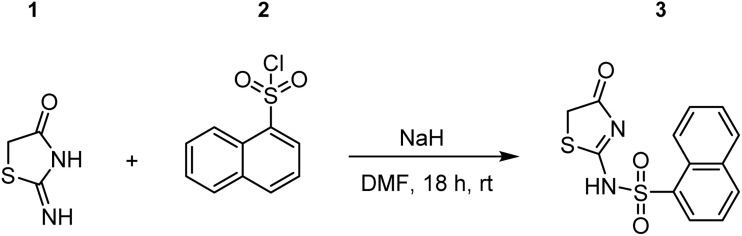
Synthesis of commercially unavailable building (4-oxo-4,5-dihydrothiazol-2-yl)naphthalene-1-sulfonamide (3). 1 = pseudothiohydantoin. 2 = naphthalene-1-sulfonyl chloride. DMF = dimethylformamide.

The synthesis of non-commercially available starting materials was performed using a protocol which was already published.^26^ Briefly, naphthalene sulfonyl chloride was reacted with 2-iminothiazolidin-4-one and the compound isolated by filtration of the acidified suspension was used without further purification as shown in Fig. 2. The analytical data was in accordance with the published data.^26^

To a suspension of the coupling product (261 mg, 0.85 mmol, 1 eq.) in absolute ethanol (1.5 mL) 4-phenoxybenzaldehyde (202 mg, 1.02 mmol, 1.2 eq.) was added followed by one drop of benzoic acid/piperidine catalyst mixture (see below). This mixture was heated at 115 °C using 150 W microwave radiation for 30 min resulting in a brown-yellowish solution. The mixture was then allowed to cool to room temperature and subsequently cooled in an ice bath for 30 min. The resulting yellow precipitate was isolated by filtration and dissolved in the minimal amount of ethyl acetate necessary to get complete solution. The compound was purified using automated column chromatography with following settings:

Injection type/flow (liquid/40 mL min^−1^), equilibration/column type (7.0 min/40 g normal phase), total runtime without equilibration time (29 min), solvents, gradient (cyclohexane : ethyl acetate, from 0 min to 12.0 min 40% B (EE) isocratic, from 12.0 min to 17.0 min gradient to 100% B (EE), then 100% B to 29 min), UV1 (254 nm), UV2 (360 nm), UV3 (450 nm), fraction size (22 mL), fractions containing product/retention time product (2–5/about 4.0–4.5 min).

**Fig. 3:**
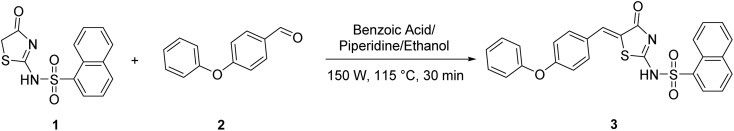
Synthesis of (*Z*)-*N*-[4-oxo-5-(4-phenoxybenzylidene)-4,5-dihydrothiazol-2-yl]naphthalene-1-sulfonamide (CSV-22). 1 = (4-oxo-4,5-dihydrothiazol-2-yl)naphthalene-1-sulfonamide; 2 = 4-phenoxybenzaldehyde; 3 = CSV-22.

The product-containing fractions were collected, the solvents were removed under reduced pressure and the resulting yellow solid was dried in high vacuum to receive the pure compound shown in Fig. 3.

Yield 29 mg (7% not optimized).

^1^H NMR (599 MHz, DMSO-*d*_*6*_) *δ* (ppm) = 7.11–7.18 (m, 4H), 7.25 (tt, *J* = 7.4, 1.1, 1H), 7.43–7.49 (m, 2H), 7.66–7.72 (m, 4H), 7.74–7.79 (m, 2H), 8.09–8.15 (m, 1H), 8.27–8.33 (m, 2H), 8.61 (dd, *J* = 8.6, 1.1, 1H), N*H* not observed, likely in deep field >10.

^13^C NMR (150 MHz, DMSO-*d*_*6*_) *δ* (ppm) = 118.4, 119.8, 120.0, 124.6, 124.6, 124.9, 127.1, 127.5, 127.6, 128.1, 128.3, 129.0, 130.3, 132.6, 133.3, 133.8, 134.7, 135.2, 155.1, 159.2, 165.6, 166.5.

HRMS: [M + Na]^+^ calcd for C_26_H_18_N_2_O_4_S_2_Na^+^ 509.06002, found 509.06009.

[2M + Na]^+^ calcd for 2C_26_H_18_N_2_O_4_S_2_Na^+^ 995.13082, found 995.13154.

*R*_f_ (ethyl acetate : cyclohexane 2 : 1; silica): 0.44.

The benzoic acid/piperidine catalyst mixture was prepared following the procedure described by McCluskey *et al.*:^26^ benzoic acid (0.61 g, 5 mmol) and piperidine (0.5 mL, 5 mmol) were dissolved in ethanol (10 mL) to afford the catalyst. The catalyst was prepared directly before use and any unused mixture was discarded.

### Lipinski rule of five

Lipinski's rule of five and drug likeliness was performed using the website: http://targetnet.scbdd.com/calcnet/index_rule/. Lipinski's rule of five is the primary selection criterion, which states that an orally active drug should satisfy the rules such as molecular mass (≤500 D), log *P* (≤5), hydrogen bond donor (≤5), hydrogen bond acceptors (≤10), and molar refractivity (40–130).^27^

### Molecular docking

We used the crystallographic structure with the PDB entry code (4G55, 4GQ1, 4I79, 1XKS, 2PM7, and 3EWE), respectively to clathrin, Nup37, Nup43, Nup133, Sec13, and Seh1. For protein preparation, the co-crystallized ligand and water molecules were eliminated except for the water molecules that were important in the interaction between the ligand and protein. By Auto Dock Tools 1.5.6 package,^28^ missing hydrogens were added. After calculating Kollman atom charges, nonpolar hydrogens were merged, and the file was saved as pdbqt. The ligand and the 3D structures of CSV-22 were depicted in Marvin Sketch Ver. 5.7, ChemAxon (http://www.chemaxon.com). The active site was predicted using the CASTp3.0 server.^29^ The docking procedure was performed by AutoDock vina.^30^ The grid box was positioned as previously described.^31^ For the binding pose model was designated the compound's conformer with the lowest binding free energy (Gibbs values, Δ*G*). The best-docked complexes were characterized and processed for further computational analysis based on binding energy values, ligand efficiency, intermolecular hydrogen bonds, and other hydrophobic and electrostatic interactions.

### Transferrin uptake assay

To evaluate clathrin-mediated endocytosis, cells were incubated for 30 minutes in DMEM without antibiotics or fetal calf serum (FCS). Following the manufacturer's protocol (Thermo Fisher Scientific), plates were placed on ice for 10 minutes to halt endocytic activity. Cells were then washed twice with ice-cold Live Cell Imaging Solution (LCIS, Cat. No. A14291DJ, Thermo Fisher Scientific) supplemented with 20 mM glucose and 1% bovine serum albumin (BSA). Fluorescent transferrin (T2871, Thermo Fisher Scientific) was added at a final concentration of 25 μg mL^−1^ in enriched LCIS. Treated groups received either Pitstop-2 or CSV-22 at 30 μM, while control groups received transferrin alone. All samples were incubated for 20 minutes at 37 °C. After incubation, cells were washed twice with LCIS and immediately imaged using confocal microscopy (Nikon Eclipse Ti2). Fluorescence was detected using excitation/emission maxima of 494/518 nm.

### Nuclear barrier permeability

The nuclear permeability assay was performed as described previously.^32^ Cells were cultured on glass-bottom Petri dishes (WillCo Wells B. V., Amsterdam, Netherlands) for imaging. Permeabilization was performed by 20 μg per mL digitonin solution in Transport Buffer (TB, 20 mM HEPES, 110 mM K-acetate, 5 mM Na-acetate, 2 mM Mg-acetate, 1 mM EGTA (pH 7.3), 2 mM DTT) for 5 minutes. Upon permeabilization, the buffer was replaced with digitonin-free TB containing 200 μg per mL 70 kDa FITC dextran (Sigma-Aldrich, Steinheim, Germany) in the control group. The treated cells received the compounds Pitstop-2 and CSV-22. Images were taken in the mid-plane of the nuclei using a Nikon Eclipse Ti2 confocal microscope at a rate of one image per minute for 30 minutes.

### Cell proliferation assay and toxicity prediction

The cell counting kit-8 (CCK-8) assay was used to determine the cell viability in cancer (A549_3R) cells, as described previously.^32,33^ The cells were cultured in a 96-well plate for 24 h at 37 °C, 5% CO_2_. Both cells (containing CCK-8 kit) were exposed for two hours to progressively increasing concentrations (1.8 μM, 3.8 μM, 7.5 μM, 15 μM, 30 μM and 60 μM) of Pitstop-2 and CSV-22. The absorbance measurement was performed at 450 nm using the plate reader, Molecular Devices. Toxicity parameters were predicted using the Tox-Prediction online tool (https://tox.charite.de/protox3/index.php?site=compound_input).^34^

### Statistical analysis

Each experimental condition was repeated at least three times. Data are presented as mean values ± standard error of the mean (S. E. M.). Results are considered statistically significant at the probability level *P* < 0.05, using GraphPad Prism 9.0.0 (GraphPad Software, Inc). The details regarding the number of experiments, analyzed cells, applied statistical tests, and *P* values are specified in the corresponding parts.

## Results and discussion

Molecular docking allows researchers to screen millions of compounds virtually, significantly reducing the time and cost associated with experimental screening.^35^

In this study, we introduced the compound CSV-22, which was synthesized and tested due to the performance initially obtained in molecular docking. Our previous work revealed that Pitstop-2 exhibits varying binding affinities to different nucleoporins (Nups) within the NPC scaffold.^31^ Specifically, Pitstop-2 showed binding affinities of −7.0 kcal mol^−1^ to Nup43, −7.5 kcal mol^−1^ to Nup37, −8.2 kcal mol^−1^ to Seh1, −7.7 kcal mol^−1^ to Sec13, and −7.9 kcal mol^−1^ to Nup133. Additionally, Pitstop-2's binding affinity to its primary target in clathrin is −8.9 kcal mol^−1^.

Our new compound demonstrated superior binding affinities to Pitstop-2, as summarized in Table 1. CSV-22 displayed binding affinities of −9.2 kcal mol^−1^ to Nup37, −7.7 kcal mol^−1^ to Nup43, −8.6 kcal mol^−1^ to Seh1, −8.5 kcal mol^−1^ to Sec13, and −8.0 kcal mol^−1^ to Nup133. It also exhibited a stronger binding affinity to clathrin (−9.8 kcal mol^−1^), suggesting potential CME inhibition. However, functional assays revealed a distinct pharmacological profile. Despite its high clathrin affinity, CSV-22 did not significantly impair transferrin uptake in NSCLC cells, indicating that CME remains largely unaffected (Fig. S1). This dissociation between clathrin binding and CME inhibition suggests that CSV-22 exerts its cytotoxic effects primarily through NPC disruption rather than endocytic blockade.

**Table 1:** Thermodynamic interaction parameters. Thermodynamic parameters (Gibbs free energy change kcal mol^−1^, Δ*G*°) obtained for the interaction of CSV-22 with the terminal β-propeller domain of the heavy chain of clathrin, and NPC scaffold Nups members of the Nup107/160 subcomplex with β-propellers (Nup37, Nup43, Seh1, Sec13, Nup133)

Binding affinity (kcal mol^−1^)		
	CSV-22	Pitstop-2
Nup37	−9.2	−7.5
Nup43	−7.7	−7.0
Seh1	−8.6	−8.2
Sec13	−8.5	−7.7
Nup133	−8.0	−7.9

**Fig. 4:**
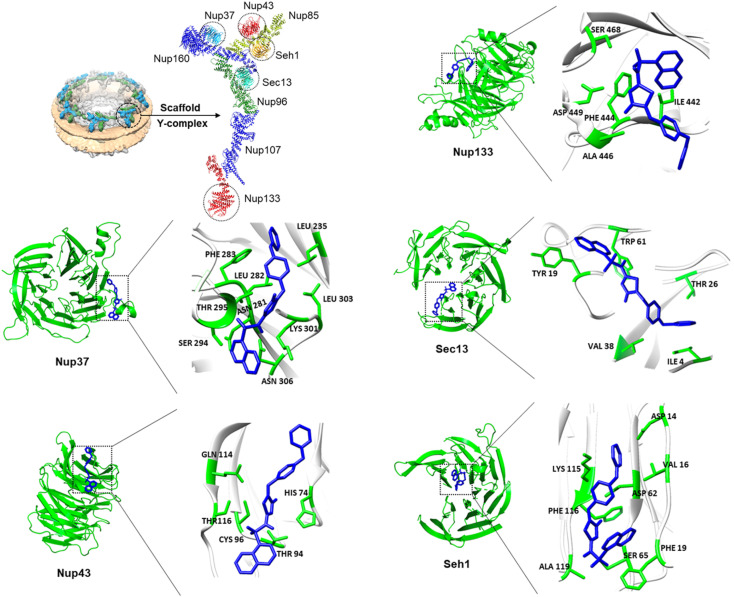
Computational docking analysis. The NPC scaffold is made up of Y-shaped subcomplexes,^9^ termed Y-complexes as shown in cryo-electron tomography analysis.^10^ CSV-22 (blue) binding to the β-propeller domains of the NPC scaffold proteins Nup37 (PDB: 4GQ1), Nup43 (PDB: 4I79), Seh1 (PDB: 3EWE), Sec13 (PDB: 2PM7), and Nup133 (PDB: 1XKS).

The interactions of the ligand, CSV-22, with the tested proteins were determined using the Protein–Ligand Interaction Profiler online tool.^36^ When we analyzed CSV-22 in clathrin, we discovered that it formed hydrogen bonds with SER67, with a bond length of 3.15 Å, a non-covalent interaction (π-interaction) with the PHE91, and hydrophobic interactions with residues ILE66 and GLN89 as seen in Fig. 4. For Nup37, CSV-22 formed hydrogen bonds with THR295 and LYS301 with bond lengths of 3.62 Å and 2.16 Å, respectively. CSV-22 also established with Nup37 hydrophobic interactions with residues LEU235, ASN281, LEU282, LEU303, ASN306 and π-interaction with PHE283 (Fig. 4).

The interactions of CSV-22 with Nup43 included hydrogen bonds with HIS74 (bond length: 3.35 Å) and THR94 (bond length: 2.31 Å) (Fig. 4). For Nup133, the compound CSV-22 formed hydrogen bonds with ALA446 (bond length: 2.17 Å), and SER468 (bond length: 1.98 Å), along with hydrophobic contacts with ILE442, and PHE444 (Fig. 4). In the nucleoporin Sec13, CSV-22 exhibited hydrophobic interaction with residues ILE4, TYR19, THR26, VAL38, and TRP61 (Fig. 4). For Seh1, CSV-22 formed a hydrogen bond with PHE116 (bond length: 3.66 Å), and ALA119 (bond length: 2.64 Å), established two hydrophobic contacts (VAL16, and PHE19), and a non-covalent molecular interaction (cation-π) with LYS115 (Fig. 4).

The docking analysis underscores the superior efficacy of CSV-22 over Pitstop-2 in binding to clathrin and nucleoporins within the NPC scaffold, which we previously determined.^31^ CSV-22 exhibits enhanced binding affinities and more extensive protein–ligand interactions, including hydrogen bonds and hydrophobic contacts, contributing to its potent inhibitory effects. The improved thermodynamic interaction parameters, particularly the Gibbs free energy changes, further highlight the stability and specificity of CSV-22 binding. These findings suggest that CSV-22's dual targeting of clathrin and the NPC could disrupt critical cellular processes more effectively than Pitstop-2, potentially leading to improved therapeutic outcomes.

Several features are implemented to determine the potential success of new drug candidates, adhering to well-established guidelines like Lipinski's Rule of Five. Lipinski's rules serve as a benchmark for evaluating the drug-likeness of compounds, focusing on key molecular properties such as molecular weight, hydrogen bond donors and acceptors, lipophilicity, and solubility.^27^

CSV-22 and Pitstop-2 were evaluated based on several critical physicochemical parameters (Table 2). CSV-22 has a total polar surface area (TPSA) of 118.51 Å^2^ and a molar refractivity (MR) of 138.8627 cm^3^ mol^−1^. It has a molecular weight of 486.56212 g mol^−1^, with one hydrogen bond donor (HBD) and seven hydrogen bond acceptors (HBA). Its log *P* value is 6.9905, which adheres to 75% of Lipinski's Rule of Five parameters, indicating a higher lipophilicity than Pitstop-2.

**Table 2:** Lipinski's rule of five for CSV-22 and Pitstop-2. Both compounds presented 75% regarding the parameters presented in the rule of five (Ro5) or Lipinski's rule of five. TPSA (Topological Polar Surface Area, Å^2^), MR (Molar Refractivity, cm^3^ mol^−1^), MW (Molecular Weight, g mol^−1^), HBD (Hydrogen Bond Donors), HBA (Hydrogen Bond Acceptors), log *P* (partition coefficient)

Molecule	TPSA	MR	MW	HBD	HBA	Log *P*	Lipinski rule of five
CSV-22	118.51	138.8627	486.56212	1.0	7.0	6.9905	75%
Pitstop-2	109.28	120.0467	473.36282	1.0	6.0	5.9607	75%

Pitstop-2 shows a slightly lower TPSA of 109.28 Å^2^ and MR of 120.0467 cm^3^ mol^−1^. Its molecular weight is 473.36282 g mol^−1^, and it has one hydrogen bond donor and six hydrogen bond acceptors. With a log *P* value of 5.9607, it also adheres to 75% of Lipinski's Rule of Five, highlighting its more favorable balance between hydrophilicity and lipophilicity.

Despite CSV-22's higher log *P* value than Pitstop-2, our data indicate superior specificity for NSCLC cells, demonstrating its potential as an adjuvant in cancer treatment. In addition, incorporating it into nanopreparations would likely improve bioavailability. Moreover, considering alternative routes of administration, such as intravenous or localized delivery methods, could bypass the limitations associated with oral administration. These strategies would allow for better targeting and efficacy, making CSV-22 a promising candidate for further development in cancer therapy.

The NPC permeability barrier is essential for maintaining cellular homeostasis by controlling access to the cell's genetic material.^37^ The integrity and tightness of this barrier are crucial for regulating nucleocytoplasmic transport, which impacts numerous cellular processes, including gene expression and signal transduction.^38^ Understanding the factors influencing NPC permeability is vital for elucidating its role in normal cellular functions and pathological conditions, such as cancer, where NPC alterations can contribute to disease progression.^39^

Recent studies have highlighted the role of NPC in cancer biology, demonstrating that dysregulation of nucleocytoplasmic transport can promote tumor progression through the mislocalization of key regulatory proteins and altered gene expression profiles.^40,41^

**Fig. 5:**
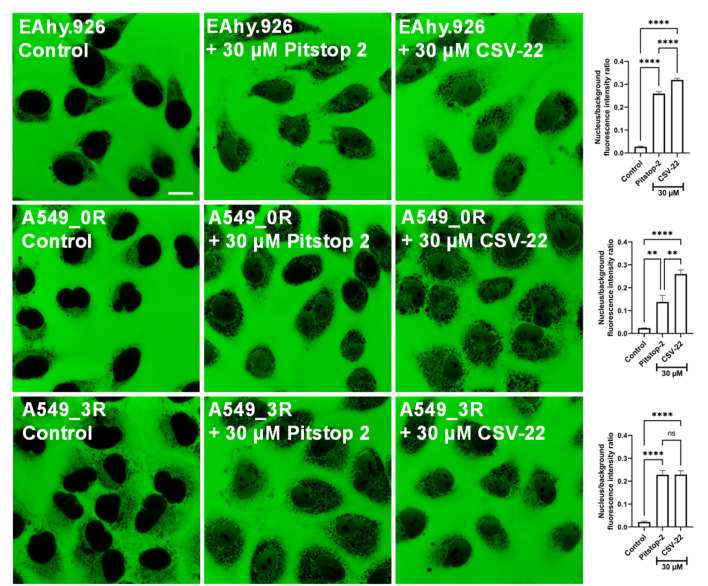
NPC barrier permeability analysis. Pitstop-2 and CSV-22 effects on the NPC permeability barrier of non-small cell lung cancer cells (A549_3R). Confocal microscopy images of A549_3R clearly show that the large 70 kDa FITC-dextran macromolecule is completely excluded from the cell nucleus in the untreated control. A549_3Rs exposed for 30 minutes to Pitstop-2 and CSV-22 exhibit green coloration inside the nucleus, indicating the entry of 70 kDa FITC-dextran and disruption of the NPC selective barrier. Each bar in the graphs corresponds to one of the experimental groups depicted in the confocal images on the left, representing the quantified nucleus/background fluorescence intensity ratio (*P* < 0.05, Two-Way ANOVA followed by the Bonferroni multiple comparison test, *N* = 3). Scale bar 10 μm.

CSV-22 disrupts of the NPC barrier in highly metastatic NSCLC cells, as shown in Fig. 5. This disruption compromised the NPC's ability to exclude large macromolecules like 70 kDa FITC-dextran, allowing these molecules to permeate the nucleus, turning the cell nucleus green. Similar effects were obtained with Pitstop-2, serving as a comparative baseline (Fig. 5). This method effectively demonstrates the activity of the cellular NPC by highlighting its permeability to large molecules. Usually, the NPC regulates the nucleocytoplasmic transport between the nucleus and cytoplasm, controlling the exchange of proteins, RNAs, and other molecules. By compromising this barrier, CSV-22 disrupts regular nucleocytoplasmic transport, potentially impeding critical cellular functions, such as transporting regulatory proteins and exporting mRNA. These disruptions can significantly affect cell viability and function, particularly in cancer cells, where altered NPC function can affect cell proliferation and survival.

**Fig. 6:**
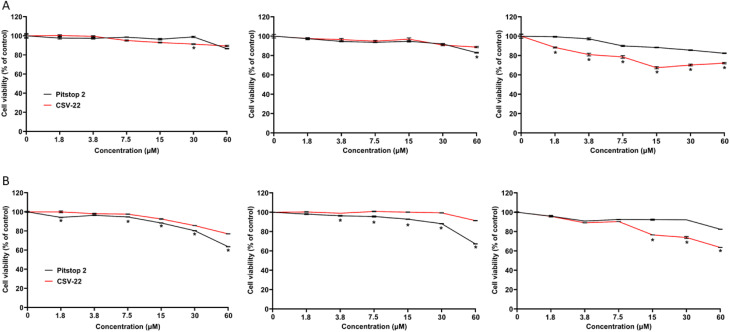
Comparative cell viability analysis. Cell viability was assessed in EA.hy926 cells (left), lowly metastatic (A549_0R, center) and highly metastatic (A549_3R, right) NSCLC cells post-2-hour (A) and 24-hour (B) exposure to escalating concentrations of Pitstop-2 and CSV-22 using the CCK-8 assay. Asterisks indicate statistically significant differential viability responses (*P* < 0.05, Two Way ANOVA followed by Bonferroni multiple comparison test). *N* = 3.

Assessing of cell viability in cancer cells is a fundamental step in evaluating the cytotoxic potential of novel therapeutic compounds. We evaluate the efficacy of CSV-22, focusing on highly metastatic NSCLC cells (A549_3R) to underscore the compound's potential in treating aggressive cancer types. CSV-22 demonstrated superior efficacy in reducing cell viability, even at the lowest concentration of 1.8 μM, as shown in Fig. 6A. Specifically, CSV-22 reduced A549_3R viability by approximately 20% at a concentration of 7.5 μM. In contrast, Pitstop-2 achieved a maximum reduction of only 12% at the concentration 60 μM.

After 24 hours of exposure, CSV-22 induced a ∼35% reduction in the viability of highly metastatic NSCLC (A549_3R), outperforming Pitstop-2, which achieved only ∼15% reduction at the tested concentration of 60 μM (Fig. 6B). Furthermore, IC_50_ analysis (Table 3) revealed that CSV-22 exhibits markedly lower IC_50_ values in A549_3R cells compared to both A549_0R and EA.hy926 controls, confirming its selective cytotoxicity toward highly metastatic NSCLC.

**Table 3:** Inhibitory concentration (IC_50_) values. IC_50_ values (μM) obtained for Pitstop-2 and CSV-22 in EA.hy926, A549_0R, and A549_3R cell lines following 2-hour and 24-hour exposure. Values reflect compound concentrations required to reduce cell viability by 50%, as determined by CCK-8 assays

Cell line	IC_50_ (2 h) CSV-22	IC_50_ (2 h) Pitstop-2	IC_50_ (24 h) CSV-22	IC_50_ (24 h) Pitstop-2
EA.hy926	>480 μM	>480 μM	254 μM	222 μM
A549_0R	>480 μM	354 μM	302 μM	227 μM
A549_3R	>480 μM	357 μM	214 μM	298 μM

Additionally, Pitstop-2 and CSV-22 showed a predicted LD_50_ of 350 mg kg^−1^ if administered orally, receiving a toxicity Class IV classification. The ProTox 3.0 web server,^34^ toxicity classes are defined according to the globally harmonized system of classification and labeling of chemicals (GHS) where the classification of compounds follows the parameters: Class I: fatal if swallowed (LD_50_ ≤ 5 mg kg^−1^), Class II: fatal if swallowed (5 mg kg^−1^ < LD_50_ ≤ 50 mg kg^−1^), Class III: toxic if swallowed (50 mg kg^−1^ < LD_50_ ≤ 300 mg kg^−1^), Class IV: harmful if swallowed (300 mg kg^−1^ < LD_50_ ≤ 2000 mg kg^−1^), Class V: may be harmful if swallowed (2000 mg kg^−1^ < LD_50_ ≤ 5000 mg kg^−1^), Class VI: non-toxic (LD_50_ > 5000 mg kg^−1^).

## Conclusion

Our study successfully introduces CSV-22 as a novel compound, designed and synthesized based on its favorable molecular docking interactions with the β-propeller domains of nucleoporins. CSV-22 presented greater lipophilicity than Pitstop-2, a parameter that can be circumvented by loading CSV-22 into nanoparticles for a controlled release at the site of action. Notably, CSV-22 showed greater cytotoxic against highly metastatic NSCLC cells, with lower IC_50_ values and pronounced effects on cell viability after 24 hours, compared to Pitstop-2. By disrupting the NPC barrier, CSV-22 interferes with nucleocytoplasmic transport, potentially undermining vital cellular processes in cancer cells. These promising findings position CSV-22 as a significant advancement in cancer therapeutics, with potential applications extending to other malignancies. Future research will focus on leveraging targeted drug delivery systems and conducting comprehensive *in vivo* studies to fully harness CSV-22's therapeutic potential and translate these findings into clinical settings.

## Author contributions

Sílvio Terra Stefanello: conceptualization; data curation; formal analysis; investigation; methodology; writing – original draft. Caren Rigon Mizdal: investigation; data curation; methodology; visualization. Christian Paul Konken: methodology; chemical synthesis; resources. Günter Haufe: conceptualization; supervision; methodology; resources. Victor Shahin: conceptualization; project administration; supervision; validation; methodology; resources; writing – review & editing.

## Conflicts of interest

The authors declare no competing financial interest.

## Supplementary Material

NA-OLF-D5NA00410A-s001

## Data Availability

The data used in the article are original and the data that supports the findings of this study are available from the corresponding author upon reasonable request. Supplementary information is available. See DOI: https://doi.org/10.1039/d5na00410a.
