# Supplementary material for: A Pitstop-2 analog impairs viability of aggressive lung cancer cells by disrupting nuclear pore integrity
Source: Nanoscale Adv. 2025 Sep 16;7(21):7040–8. doi: 10.1039/d5na00410a (PMC12465235; doi:10.1039/d5na00410a)
Supplement: NA-007-D5NA00410A-s001 [file NA-007-D5NA00410A-s001.pdf]

Supplementary Information (SI) for Nanoscale Advances.
This journal is © The Royal Society of Chemistry 2025

Supplementary Material

A Pitstop-2 analog impairs viability of aggressive lung cancer cells by disrupting nuclear pore integrity

Sílvio Terra Stefanello[1‡], Caren Rigon Mizdal[1‡], Christian Paul Konken[2,3], Günter Haufe [4], Victor Shahin[1]**

[1] University of Münster, Institute of Physiology II, Robert-Koch-Str. 27b, 48149 Münster, Germany.

[2] University of Münster, European Institute for Molecular Imaging (EIMI), Münster, Germany.

[3] University Hospital Münster, Department of Nuclear Medicine, Münster, Germany.

[4] University of Münster, Organic Chemistry Institute, Münster, Germany.

‡Equally contributed

*** Corresponding Author:**

Dr. Silvio Terra Stefanello
E-mail: terraste@uni-muenster.de

Prof. Dr. Victor Shahin
E-mail: shahin@uni-muenster.de

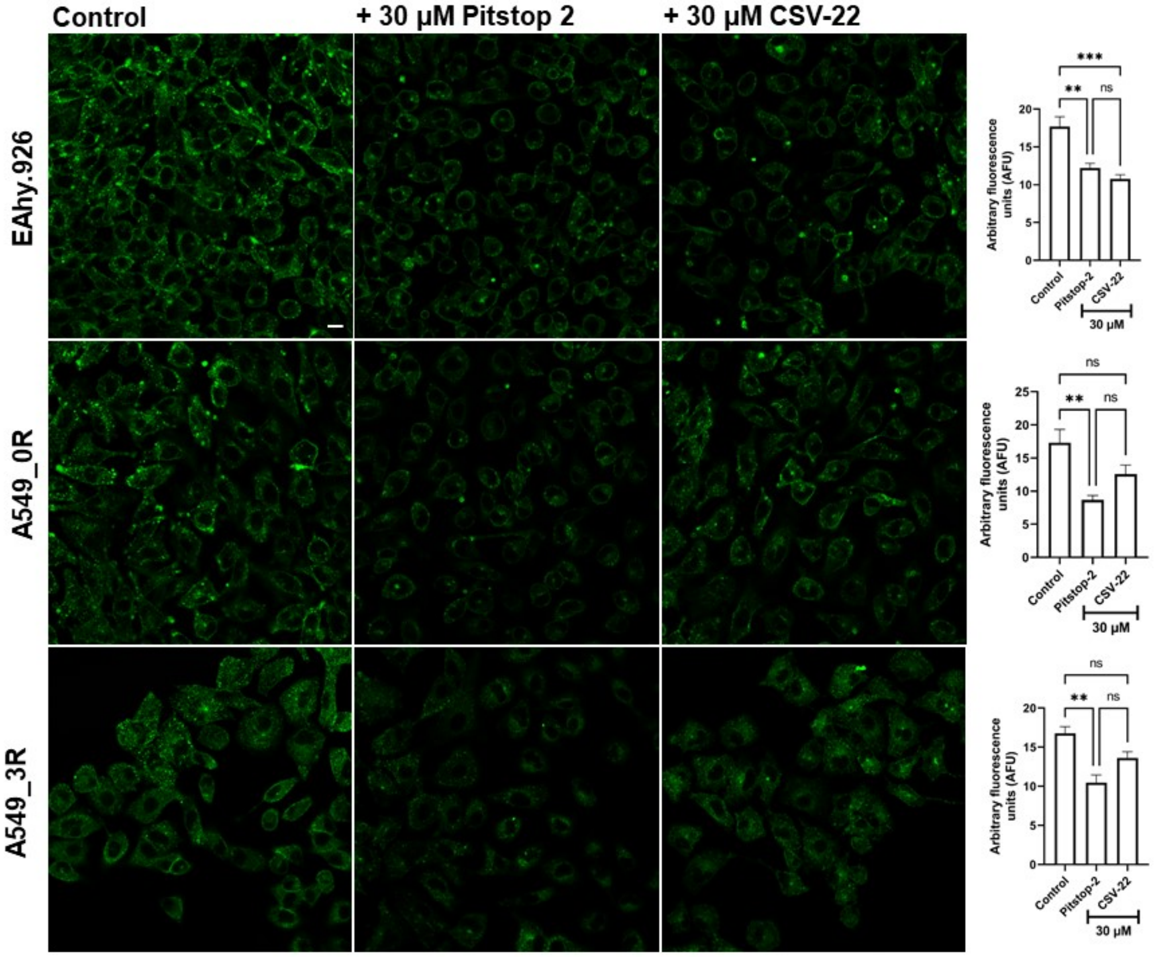

Figure S1. Effects of Pitstop-2 and CSV-22 on clathrin-mediated endocytosis in EA.hy926, A549_0R, and A549_3R cells. Confocal microscopy images show the internalization of fluorescently labeled transferrin after 30 minutes of exposure to each compound. The bar graphs on the right represent the quantified mean fluorescence intensity per 10 cells ($P < 0.05$, one-way ANOVA followed by Bonferroni multiple comparison test, $N = 4$). Scale bar: 10 µm.
